# Physiologically Based Pharmacokinetic Modeling of Cefadroxil in Mouse, Rat, and Human to Predict Concentration–Time Profile at Infected Tissue

**DOI:** 10.3389/fphar.2021.692741

**Published:** 2021-12-23

**Authors:** Zhongxia Tan, Youxi Zhang, Chao Wang, Le Sun

**Affiliations:** ^1^ Department of Pharmaceutics, School of Pharmacy, China Medical University, Shenyang, China; ^2^ Department of Pharmacy, The Fourth Affiliated Hospital, China Medical University, Shenyang, China; ^3^ Liaoning Inspection Examination and Certification Centre, Shenyang, China

**Keywords:** PBPK model, cefadroxil, hPept1, Kp, species extrapolation

## Abstract

The aim of this study was to develop physiologically based pharmacokinetic (PBPK) models capable of simulating cefadroxil concentrations in plasma and tissues in mouse, rat, and human. PBPK models in this study consisted of 14 tissues and 2 blood compartments. They were established using measured tissue to plasma partition coefficient (*K*_p_) in mouse and rat, absolute expression levels of hPEPT1 along the entire length of the human intestine, and the transporter kinetic parameters. The PBPK models also assumed that all the tissues were well-stirred compartments with perfusion rate limitations, and the ratio of the concentration in tissue to the unbound concentration in plasma is identical across species. These PBPK models were validated strictly by a series of observed plasma concentration–time profile data. The average fold error (AFE) and absolute average fold error (AAFE) values were all less than 2. The models’ rationality and accuracy were further demonstrated by the almost consistent *V*_ss_ calculated by the PBPK model and noncompartmental method, as well as the good allometric scaling relationship of *V*_ss_ and *CL*. The model suggests that hPEPT1 is the major transporter responsible for the oral absorption of cefadroxil in human, and the plasma concentration–time profiles of cefadroxil were not sensitive to dissolution rate faster than T_85%_ = 2 h. The cefadroxil PBPK model in human is reliable and can be used to predict concentration–time profile at infected tissue. It may be useful for dose selection and informative decision-making during clinical trials and dosage form design of cefadroxil and provide a reference for the PBPK model establishment of hPEPT1 substrate.

## Introduction

Cefadroxil, a first-generation cephalosporin, has been commonly used in the treatment of different kinds of infections including urinary tract, skin, and respiratory infections (Tanrisever and Santella, 1986). Cefadroxil has a high bioavailability (Santella and Henness, 1982) despite its poor lipophilicity. It is a substrate of the peptide transporter PEPT1, and PEPT1 plays an important role in its intestinal absorption (Posada and Smith, 2013b). It is minimally metabolized in the body and excreted primarily by the kidney, with over 90% of the administered dose being recovered in the urine intact within 24 h (Nightingale, 1980). The distribution of cefadroxil in the infected tissue is directly related to its pharmacological effects. Some tissue distribution data in human have been published (Nightingale, 1986; Akimoto et al., 1994; Nungu et al., 1995). However, they were only the concentration ratios of tissue/serum (plasma) at the peak time or other one time point. This did not reflect well the distribution of the drug in tissues.

The physiologically based pharmacokinetic (PBPK) model, a mechanistic quantitative framework, is established mainly based on physiological organ sizes, blood flow rates, tissue to plasma partition coefficient (*K*_p_), elimination mechanisms, etc. (Kesisoglou et al., 2016). It can provide insight regarding drug concentration–time profiles in various tissues and build the relationship between dose and pharmacokinetic (PK) profiles in specific tissues to further assess the pharmacological effects. A number of PBPK models have been developed for assessing the clinical relevance of concentrations at target tissues, pharmacokinetic and pharmacodynamic relationship, *in vivo* pharmacokinetic properties of nanoparticles, the drug–drug interaction, etc. (Jones et al., 2015; Li et al., 2017). However, building an exact PBPK model needs to measure or calculate drug concentrations in various tissues, and this kind of data is very difficult to obtain in human.

*K*_p_ is an important parameter in the PBPK model. *K*_p_ in human can be obtained by *in silico* method, such as Poulin method (Poulin and Theil, 2002), Berezhkovskiy method (Berezhkovskiy, 2004), and Rodgers and Rowland method (Rodgers et al., 2005). The tissues and plasma are assumed to be a combination of water, lipids, and proteins at pH = 7.4. *K*_p_ of tissues is mainly calculated based on the volume fraction of lipid, phospholipid, and water in tissue, unbound fraction of drug in tissue and plasma, and octanol/water partition coefficient (log *P*). *In silico* method can obtain the *K*_p_ values conveniently and quickly. However, it cannot consider the active diffusion of some drugs, for example, the distribution of the influx and efflux transporter involved.

*K*_p_ also can be measured in animals, such as mouse and rat, then extrapolated to the *K*_p_ in human. A PBPK model describing and predicting terbinafine concentration in plasma and tissues in humans was established by supposing the identical *K*_p_ among mammals (Hosseini-Yeganeh and McLachlan, 2002). Chen et al. (2016) assumed that the ratio of the concentration in tissue to the unbound concentration in plasma is identical across species and then calculated the *K*_p_ in mouse, monkey, dog, and human based on *K*_p_ in rat and unbound fraction of deoxypodophyllotoxin in plasma of the corresponding species and rat. A PBPK-pharmacodynamic (PD) model linking to stomach to simultaneously predict vonoprazan PK and its antisecretory effects following administration to rats, dogs, and humans was developed (Kong et al., 2020). Due to a large species difference in free fraction of vonoprazan in the plasma (*f*_u_), the *K*_p_ in dog and *K*_p_ in human were calculated by *f*_u_ (dog/human) × *K*_p_,rat/*f*_u_,_rat_ based on the assumption that the ratios of tissue to plasma free concentration were identical across species (Kong et al., 2020), just like what Chen et al. (2016) did in their study.

Therefore, in this study, PBPK models were established to simulate the plasma and tissue concentration–time profiles in mouse and rat after intravenous injection of cefadroxil using the measured *K*_p_ in mouse and rat. After validating these models by *in vivo* data, a PBPK model in human was established to simulate the plasma and tissue concentration–time profile after oral administration using the *K*_p_ extrapolated from mouse and rat, as well as the absolute expression levels of hPEPT1 along the entire length of the human intestine and the transporter kinetic parameters.

## Methods

### The Establishment of Physiologically Based Pharmacokinetic Models in Mouse, Rat, and Human

**FIGURE 1:**
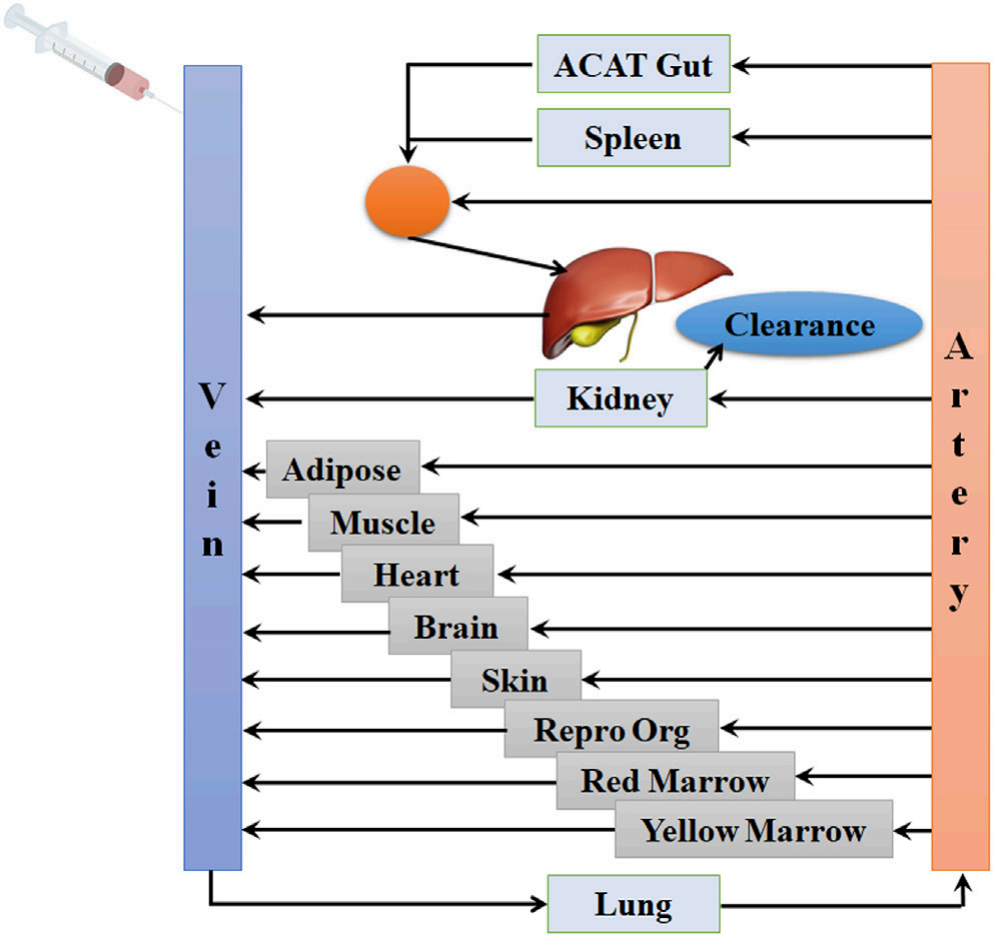
The model structure of the physiologically based pharmacokinetic (PBPK) model in mouse and the PBPK model in rat after intravenous injection of cefadroxil solution.

**FIGURE 2:**
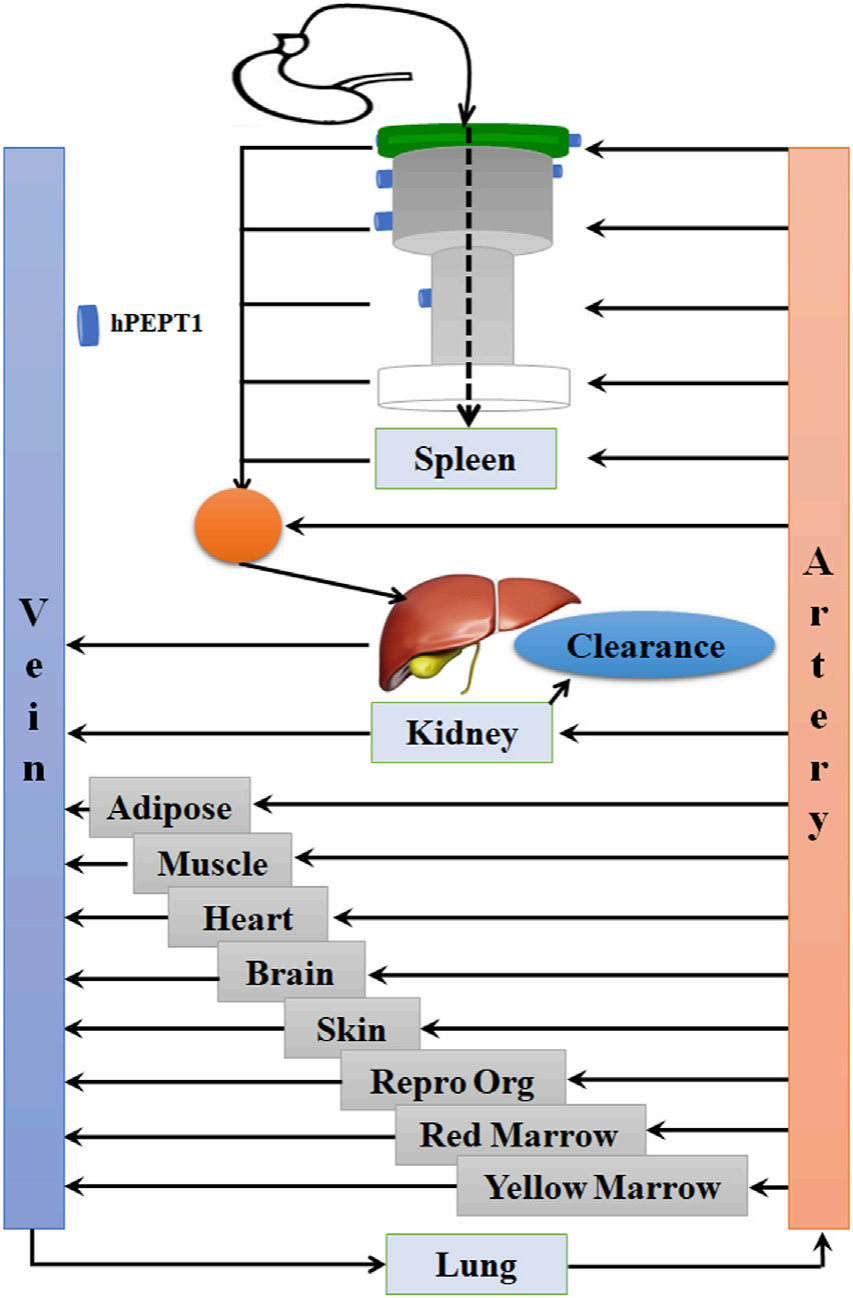
The model structure of the physiologically based pharmacokinetic (PBPK) model in human after oral administration of cefadroxil tablet.

The PBPK model in mouse and PBPK model in rat simulating the plasma and other tissue concentration–time profiles after intravenous injection of cefadroxil solution were established, respectively. The PBPK model in human simulating the plasma and other tissues concentration–time profiles after oral administration of cefadroxil tablet was established based on the established PBPK models in mouse and rat. The PBPK models in mouse, rat, and human were established using GastroPlus™ (version 9.7, Simulation Plus, Lancaster, CA, USA). The default mouse, rat, and human fasted physiological models provided in GastroPlus™ were used. The model structure of PBPK model in mouse and PBPK model in rat after intravenous injection of cefadroxil solution was shown in Figure 1. The model structure of PBPK model in human after oral administration of cefadroxil tablet was shown in Figure 2. The most common indications for cefadroxil are infection in the urinary tract and skin. The skin and kidney were both included in our models. The physiological parameters of PBPK models were shown in Table 1. The basic physicochemical and biopharmaceutical properties used in PBPK models were shown in Table 2. Over 90% of the cefadroxil is excreted unchanged in the urine within 24 h. Thus, the kidney was designed as the elimination organ.

**TABLE 1:** Physiological parameters of PBPK models in mouse, rat, and human.

	Mouse		Rat		Human	
Tissues	Volume (ml)	The blood flow (ml/s)	Volume (ml)	The blood flow (ml/s)	Volume (ml)	The blood flow (ml/s)
Lung	0.15833	0.11347	2.604	0.9389	1,125.5	98.1779
Arterial supply	0.57	0.11347	6.944	0.9389	2,148.17	98.1779
Venous return	1.13	0.11347	14.012	0.9389	4,296.34	98.1779
Adipose	1.91048	0.00127	12.4	0.00784	24,307.3	8.09433
Muscle	9.2219	0.01517	151.28	0.14695	25,174.7	12.5874
Liver	1.66355	0.03352	12.772	0.23111	1,590.1	24.3444
ACAT gut	—	0.02498	—	0.14689	—	12.263
Spleen	0.10081	0.0015	0.744	0.01175	169.973	2.83294
Heart	0.10922	0.00467	1.488	0.07638	337.26	4.10344
Brain	0.41647	0.00759	1.53402	0.02554	1,493.16	12.6919
Kidney	0.38929	0.0213	4.588	0.18019	360.411	22.1051
Skin	3.51582	0.01009	49.6	0.11361	2,831.73	5.66346
ReproOrg	0.148	0.00049	3.1	0.00979	50.6174	0.17716
Red marrow	0.83204	0.01355	2.31146	0.03568	1,106.48	5.53242
Yellow marrow	0.52449	0.00085	5.14347	0.00794	3,075.64	1.53782
RestOfBody	1.3735	0.00497	30.282	0.10386	2,681.07	1.34054

**TABLE 2:** Physicochemical and biopharmaceutical properties of cefadroxil used in the PBPK models.

Input parameter	PBPK model in mouse	PBPK model in rat	PBPK model in human
Molecular weight	363.39^a^		
Log *P*	−0.4^a^		
p*K*_a_	9.71 (acid), 7.21 (base), 2.55 (acid)^b^		
Solubility (mg/ml @pH = 5.15)	12.44^c^		
Blood/plasma conc. ratio	1		
Use Exp Plasma Fup (%)	71.9^a^		
Mean precipitation time (s)	900^d^		
Diffusion coefficient (cm^2^/s × 10^5^)	0.75^d^		
Drug particle density (g/ml)	1.2^d^		
Dose (mg)	0.1, 0.4, 4.79	0.62	375.5, 1,126.5, 2,253	
Formulation option	IV: Bolus	IV: Bolus	IR: Suspension	
Passive P_eff_ (cm/s × 10^4^)			0.03^d^	
*V*_ss_ (L)	0.012	0.159	24.747	
Clearance (L/h)	Kidney: 0.031^e^	Kidney: 0.18^e^	Kidney: 8.50^e^	

a Drugbank.

b Shalaeva et al., 2008.

c Shoghi et al., 2013.

d Default value in GastroPlus™.

e Optimizing value based on observed plasma concentration–time curve.

The *K*_p_ values of lung, adipose, muscle, liver, spleen, heart, brain, kidney, skin, reproductive organ, red bone marrow, yellow bone marrow, and rest-of-body were shown in Table 3. *K*_p_ is one important parameter for PBPK model and reflects the distribution of the drug in various tissues. However, the *K*_p_ of human is difficult to obtain. Thus, it is commonly extrapolated from experimental animals. The ratio of the concentration in tissue to the unbound concentration in plasma is assumed identical across species. No literature published that there is a large species difference in free fraction in the plasma for cefadroxil. Thus, in this study, the *K*_p_ values in mouse, rat, and human are assumed to be the same. The observed *K*_p_ values in mice (Posada and Smith, 2013a) were measured by *C*_tissue_/*C*_plasma_. The *C*_tissue_ and *C*_plasma_ were collected at 20 min after 178 nmol/g oral doses of cefadroxil, since this time best represented the *T*_max_ of cefadroxil. The observed *K*_p_ values in rat were measured by *AUC*_0-∞, tissue_/*AUC*_0-∞, plasma_ (Esumi et al., 1979) and *C*_tissue_/*C*_plasma_ in steady state (Kim et al., 2014). The mean or median *K*_p_ values of lung, muscle, liver, spleen, heart, brain, and kidney in mice and rats were used in the PBPK models. The predicted *K*_p_ values were used for skin, red marrow, and yellow marrow. The *K*_p_ values of reproductive organ and rest of body were supposed as 0.5; the *K*_p_ value of adipose was supposed as 0.1 (Table 3).

**TABLE 3:** Summary of cefadroxil *K*_p_ values.

Tissue	Predicted *K*_p_ values by lukacova (Rodgers-single) method using GastroPlus™	Observed *K*_p_ values in mice (Posada and Smith, 2013a)	Observed *K*_p_ values in rats (Esumi et al., 1979)	Observed *K*_p_ values in rats (Kim et al., 2014)	The *K*_p_ values used in PBPK models
Lung	1.71	0.60	0.44		0.52
Adipose	0.25				0.1
Muscle	0.87	0.32	0.18		0.25
Liver	1.82	2.14	0.81	0.37	0.81
Spleen	1.42	0.35	0.47		0.41
Heart	1.17	0.20	0.24	0.24	0.23
Brain	0.54		0.11	0.12	0.11
Kidney	2.01	7.03	6.72	15.4	6.87
Skin	0.89				0.89
Reproductive organ	2.02				0.5
Red marrow	0.47				0.47
Yellow marrow	0.25				0.25
Rest of body	1.43				0.5

About the PBPK model in human, the absorption scale factors (ASFs) calculated by Opt logD Model SA/V6.1 were used. The absolute expression contents of hPEPT1 in duodenum, jejunum 1, jejunum 2, ileum 1, ileum 2, and ileum 3 along the entire length of the human intestine were 16.28, 87.84, 73.93, 78.61, 63.41, and 49.29 mg, respectively (Groer et al., 2013; Drozdzik et al., 2014; Sun et al., 2018). These values were measured by liquid chromatography-tandem mass spectrometry (LC-MS/MS) and had been successfully used in our other PBPK model for valacyclovir (Sun et al., 2018). The hPEPT1 expressions of the cecum and colon were adjusted to zero. The values of *K*_m_ and *V*_max_ were 860.31 mg/L and 0.0025 μg/s/mg-hPEPT1, respectively. They are the values corresponding to the hPEPT1-mediated transport of cefadroxil inputted into the model. The *K*_m_ was measured by *in situ* single-pass intestinal perfusion in huPepT1 mice with cefadroxil concentration in perfusate buffer that varied from 0.01 to 25 mM in the literature (Hu and Smith, 2016). The *V*_max_ (0.056 ± nmol/s/cm^2^) was optimized when establishing the model.

### Model Validation

The PBPK model in mice was used to simulate the plasma concentration–time profiles following intravenous injection of 11, 44.5, and 528 nmol/g cefadroxil. Then, the simulated profiles were validated by the observed concentration–time profiles (Posada and Smith, 2013a; Hu and Smith, 2016). The observed data were obtained by the following method. The wild-type mice were given 11, 44.5, and 528 nmol/g body weight (*BW*) [^3^H]cefadroxil by intravenous bolus injection. Serial blood samples were collected at 1, 2.5, 5, 10, 20, 30, 45, 90, and 120 min after dosing *via* tail transections. The cefadroxil in the plasma samples was measured using a dual-channel liquid scintillation counter (Posada and Smith, 2013a; Hu and Smith, 2016).

The PBPK model in rat was used to simulate the plasma concentration–time profiles of cefadroxil following intravenous injection of 2 mg/kg. Then, the simulated profile was validated by the observed concentration–time profile (Jin et al., 2014; Kim et al., 2014). The observed data were obtained by the following method. The rats were administered intravenous cefadroxil (2 mg/ml in phosphate-buffered saline) at a dose of 2 mg/kg. Blood samples were taken from the femoral artery cannula at 0, 5, 15, 30, 60, 90, 120, 180, and 240 min after dosing. The cefadroxil in the plasma samples was measured by LC-MS/MS (Jin et al., 2014; Kim et al., 2014).

The PBPK model in human was used to simulate the plasma concentration–time profiles following oral administration of 5, 15, and 30 mg/kg cefadroxil. Then, the simulated profiles were validated by the observed concentration–time profiles (Garrigues et al., 1991). The observed data were obtained by the following method. Cefadroxil was given orally to the 6 volunteers in doses of 5 and 15 mg/kg. Three of the volunteers also received 30 mg/kg. Blood was taken at 0, 10, 20, 30, 45, 60, 75, 90, 120, 180, 240, 360, and 480 min after dosing. Cefadroxil was assayed by high-performance liquid chromatography within 4 days (Garrigues et al., 1991).

Cefadroxil is well absorbed on oral administration; the fraction of cefadroxil dose absorbed was nearly 100%. In addition, the elimination phase of oral administration and intravenous injection should be consistent. Thus, concentrations of cefadroxil in plasma, kidney, muscle, spleen, heart, lung, liver, and brain of rats following 25 and 100 mg/kg intravenous injection were simultaneously predicted, and the prediction was validated by observed data at 1 and 3 h in rats after oral administration of 25 and 100 mg/kg (Esumi et al., 1979). Simulating the oral absorption process requires membrane permeability in rat and others’ data; this brings a lot of uncertainties to the PBPK model. The establishment of the PBPK model in rats in this study is to provide reliable distribution and elimination data for the human PBPK model. The intravenous injection model is conducive to obtaining accurate distribution and elimination data. Therefore, the PBPK model in rat was established to simulate intravenous injection, and the prediction was validated by the observed data at the elimination phase.

The fold error (FE), average fold error (AFE), and absolute average fold error (AAFE) were calculated as follows:

$$\mathrm{FE}=\frac{Predicted_{i}}{Observed_{i}}$$ (1)

$$\mathrm{AFE}=10^{\frac{1}{n}*\sum \log\left(\left|\frac{Predic\mathrm{te}d_{i}}{Observed_{i}}\right|\right)}$$ (2)

$$\mathrm{AAFE}=10^{\frac{1}{n}*\sum \left|\log\left(\frac{\mathrm{Pr}edicted_{i}}{Observed_{i}}\right)\right|}$$ (3)

where Predicted_*i*_ is the predicted concentration at time point *i*, Observed_*i*_ is the observed concentration at time point *i*, and n is the number of time points at which the concentration was determined. The FE indicates the predictive accuracy of each data point, as shown in Eq. 1. The AFE indicates whether the predicted profile underestimates or overestimates the observed values, as shown in Eq. 2. The AAFE quantifies the absolute error from the observed values, as shown in Eq. 3. If the FE of all the data points is between 0.3 and 3 (within 3-fold error) and the AFE and AAFE are both less than 2, it can be considered as a successful simulation.

### Ethics

The *in vivo* human data and animal data used in this article were all cited from references. Thus, the ethical review process was not needed in this study and was not provided.

### Parameter Sensitivity Analysis

Parameter sensitivity analysis (PSA) was performed using PBPK model in human to find out the key factors that influenced the simulated cefadroxil plasma concentration–time profile. The initial input values were varied in the range of 0.1–10 times to allow 10-fold range increase and decrease. *C*_max_ and *AUC*_0-∞_ of cefadroxil were then evaluated at each of the input values for each of the parameters studied.

### Model Application

The PBPK model in human was used to quantitatively evaluate the effect of hPEPT1 on the oral absorption of cefadroxil after its predictive performance had been fully validated. The fraction of cefadroxil dose absorbed was simulated in both the absence and presence of hPEPT1 expression in the intestine.

The validated PBPK model in human was also used to quantitatively evaluate the effect of dissolution rate on the oral absorption of cefadroxil. The simulated plasma concentration–time profiles of cefadroxil were obtained, respectively, at different dissolution rates (T_85%_ = 0.5, 1, 1.5, 2, 4, 6 h). The simulated plasma concentration–time profile obtained by input T_85%_ = 0.5 dissolution rate [>85% solubility (pH 1.2–6.8) in 0.5 h; “very rapid dissolution”] was used as the reference, then it was used to compare with those obtained by inputting other dissolution rates.

## Results

### The Physiologically Based Pharmacokinetic Models in Mouse, Rat, and Human

**FIGURE 3:**
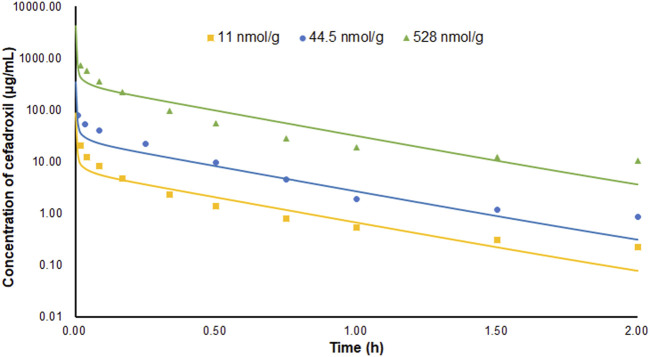
Predicted and observed plasma concentration–time profiles of cefadroxil in mice after intravenous injection of 11, 44.5, and 528 nmol/g (n = 6–8).

**FIGURE 4:**
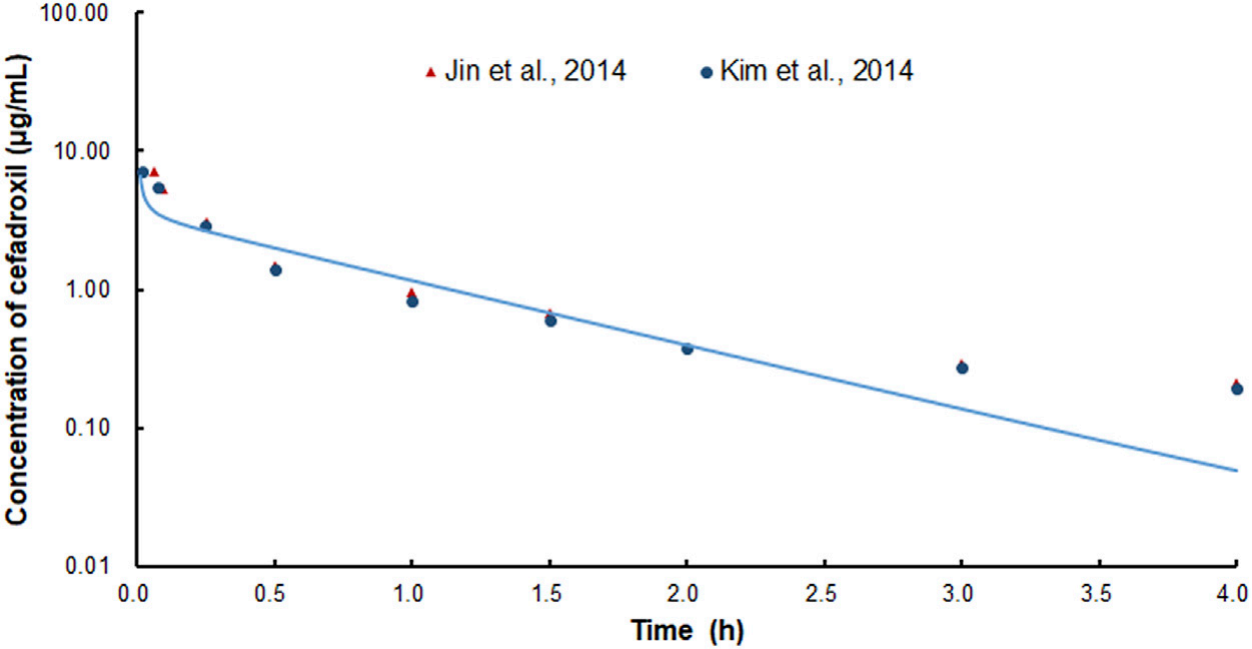
Predicted and observed plasma concentration–time profiles of cefadroxil in rats after intravenous injection of 2 mg/kg (n = 4–5).

**FIGURE 5:**
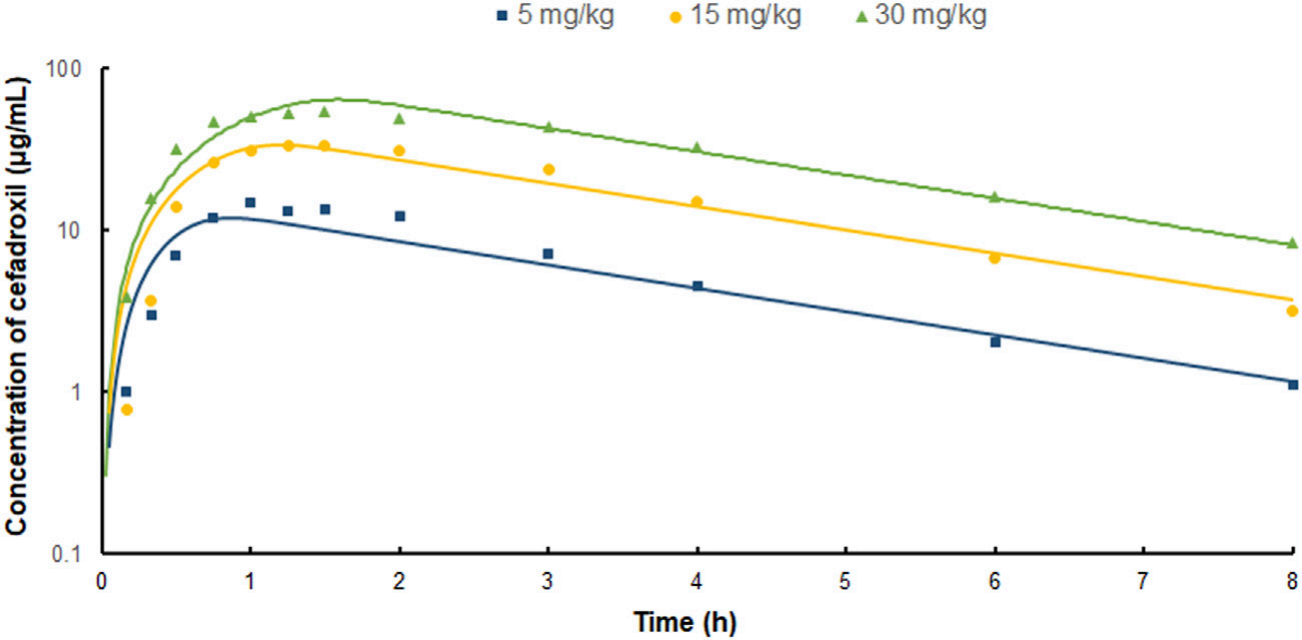
Predicted and observed plasma concentration–time profiles of cefadroxil in human after oral administration of 5, 15, and 30 mg/kg (n = 3).

**FIGURE 6:**
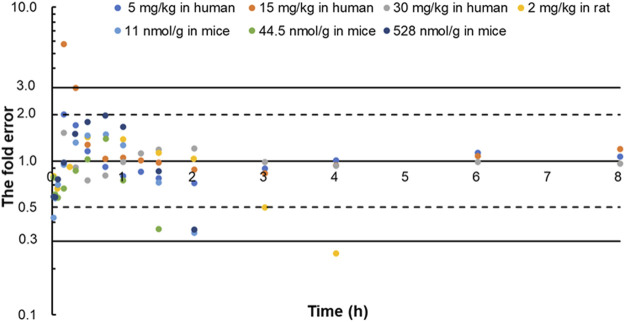
The fold error of all the predicted and observed concentration points in Figure 3, Figure 4, and Figure 5. They were calculated by Predicted_*i*_/Observed_*i*_. These data should be within 3-fold error.

The predicted and observed plasma concentrations of cefadroxil obtained from the PBPK models in mouse, rat, and human are present in Figure 3, Figure 4, and Figure 5. As shown in the above Figures, the observed and predicted plasma concentration–time profiles superimposed. The predicted and observed values of *C*_max_ and *AUC*_0-∞_ after each dose administration of cefadroxil to mouse, rat, and human are shown in Table 4. The predicted values and observed values are close. The models in mouse, rat, and human were all validated by FE. Most of the FE values were within 2-fold error (Figure 6), but there were two outliers. The FE value of data point at 0.17 h of model in human simulating 15 mg/kg was without 3-fold error (FE = 5.76). The FE value of data point at 4 h of model in rat was without 3-fold error (FE = 0.25). These may be caused by errors in the blood sampling or detection for the first and the last time point. The AFE and AAFE values for models in mouse, rat, and human were all less than 2. It indicates an adequate fitting, and the PBPK models in mouse, rat, and human are accurate and reliable.

**TABLE 4:** The predicted and observed values of *C*_max_ and *AUC*_0-∞_ after each dose administration of cefadroxil to mouse, rat, and human.

Species	Dose (mg)	Dosing route	*C*_max_ (μg/ml)		*AUC*_0-∞_ (μg·h/ml)	
			Predicted	Observed	Predicted	Observed
Mouse	0.1	IV			3.17	3.79
	0.4	IV			12.69	18.91
	4.79	IV			151.98	169.97
Rat	0.62	IV			3.42	4.04
Human	375.5	Oral	12.07	14.69	44.14	49.15
	1,126.5	Oral	34.28	33.88	132.37	133.96
	2,253	Oral	64.43	53.82	264.50	264.5

**FIGURE 7:**
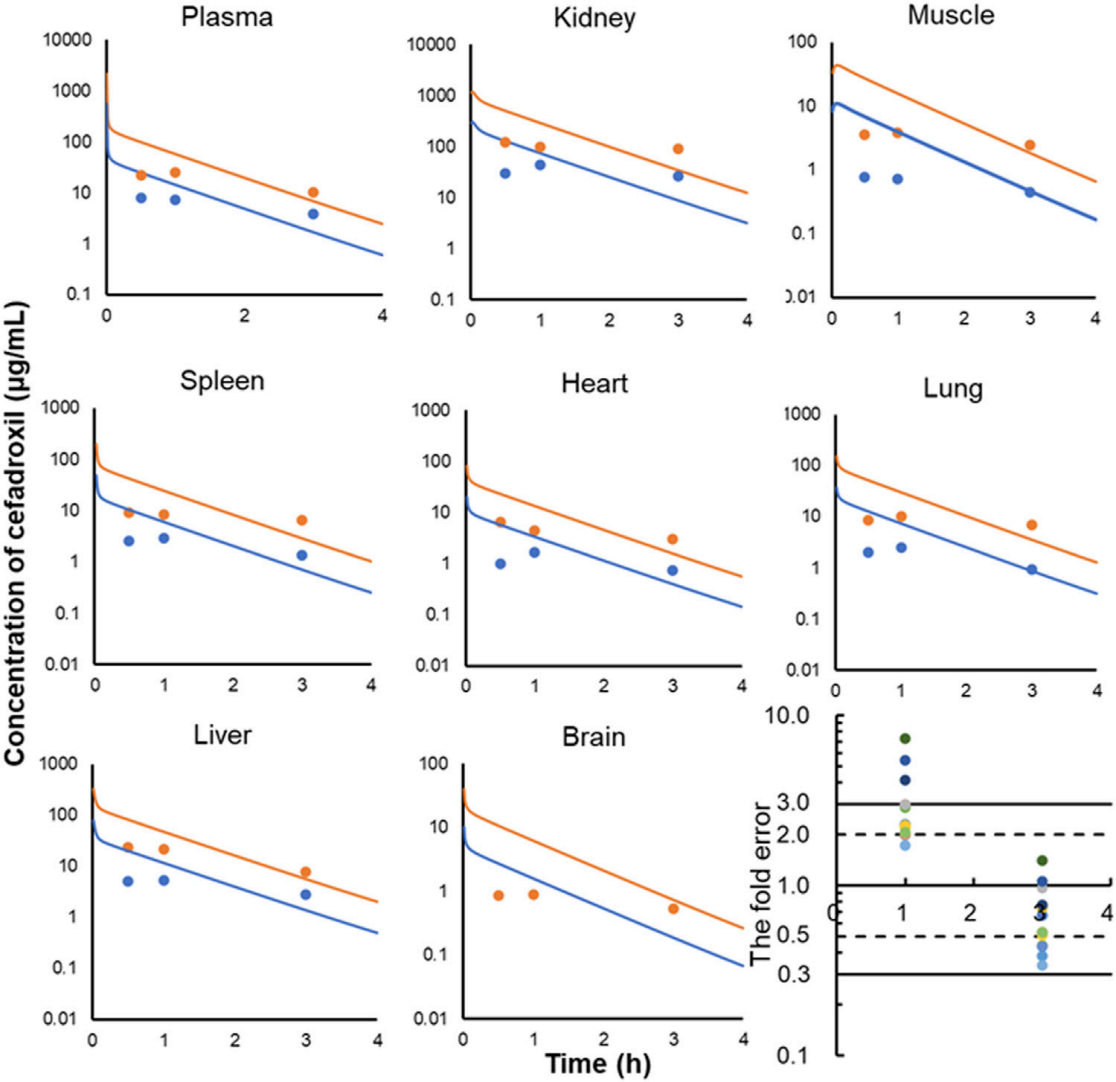
Observed and predicted concentrations of cefadroxil in plasma **(A)**, kidney **(B)**, muscle **(C)**, spleen **(D)**, heart **(E)**, lung **(F)**, liver **(G),** and brain **(H)** of rat (25 and 100 mg/kg). The observed values were obtained by oral administration (n = 3); the predicted values were obtained by intravenous injection. All of the fold errors at 1 and 3 h in plasma and tissues **(I)**.

The predicted and observed plasma/tissue concentrations were shown in Figure 7. The predicted concentrations of plasma and tissues at 0.5 h were all higher than the observed data. This was because the administration method of prediction was intravenous injection, but observed data were obtained by oral administration. The concentrations at the elimination phase were validated by the observed value. The FEs of 1 and 3 h were shown in Figure 7I. There were three points without 3-fold error. One was the point at 1 h in brain (FE = 7.25). This may be caused by the existing blood–brain barrier for cefadroxil. There were not observed data for 25 mg/kg in brain in the literature because they were not detected. Two of them were the points of 25 and 100 mg/kg at 1 h in muscle (FE = 5.44, FE = 4.16). This may be caused by different methods of administration or other reasons.

**FIGURE 8:**
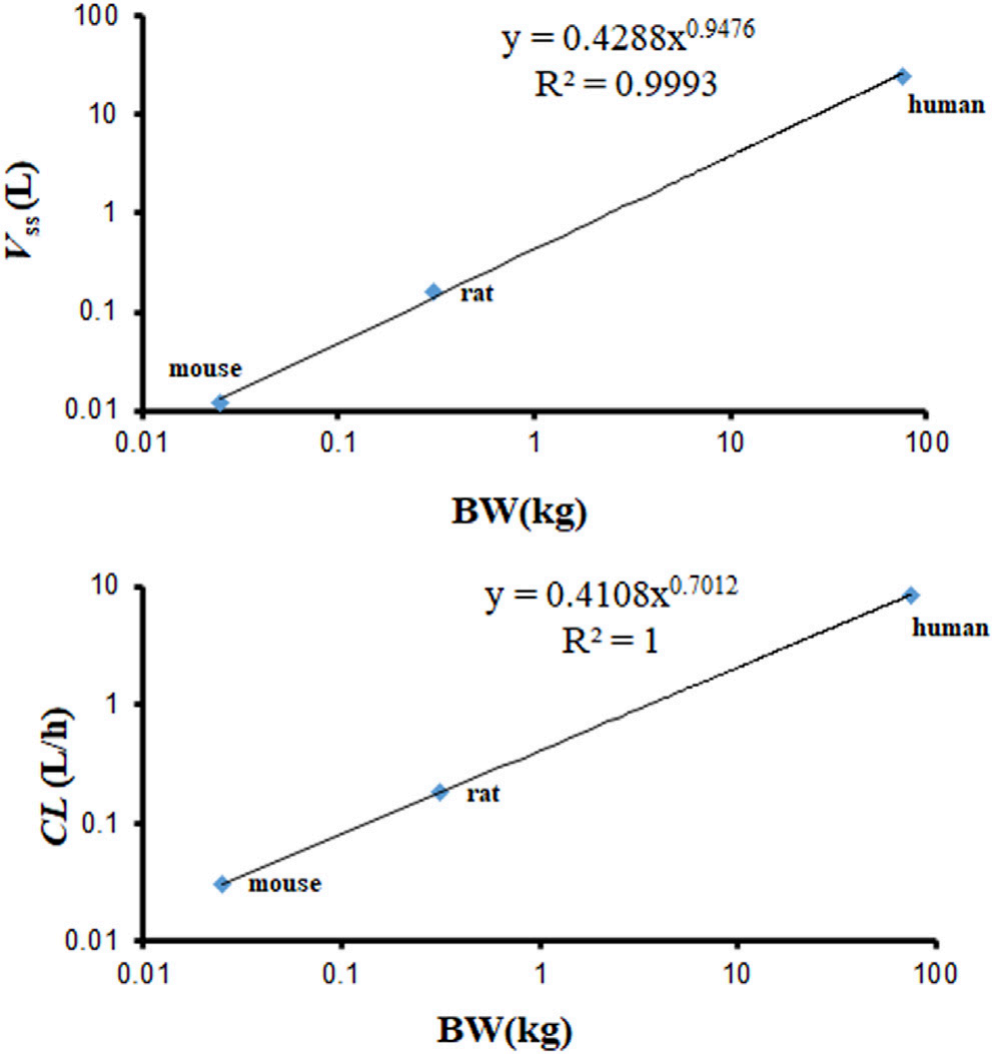
Allometric relationships for *V*_ss_ and *CL* in physiologically based pharmacokinetic (PBPK) models vs. body weight (*BW*) of various species.

The *V*_ss_ calculated by the PBPK model is based on the *K*_p_ and volume of tissues. The *V*_ss_ values of mouse, rat, and human were 0.012, 0.159, and 23.25 L. They are almost identical with the values published in the observed PK articles (0.012 L in mouse, 0.26 L in rat) (Kim et al., 2014; Hu and Smith, 2016). The *V*_ss_ and *CL* used in the PBPK model required an allometric scaling relationship with *BW* (Y = *a* × *BW* ^*b*^) (*R* ^2^ of *V*_ss_ = 0.9993, *R* ^2^ of *CL* = 1), as shown in Figure 8. The estimated slopes (a) for *V*_ss_ and *CL* were 0.4288 and 0.4108. The scaling exponent (b) for *V*_ss_ is equal to 0.9476. The value for *CL* is 0.7012. The rational *V*_ss_ and good allometric scaling relationship further verify the reliability of PBPK models in this study.

The predicted tissues and plasma concentrations of cefadroxil after oral dosing 15 mg/kg obtained from the PBPK model in human were shown in Supplementary Figure S1 and Supplementary Table S1. The *C*_max_ of lung, plasma, adipose, muscle, liver, spleen, heart, brain, kidney, and skin was 17.8, 34.23, 3.4, 8.33, 33.37, 14.05, 7.88, 3.77, 210.22, and 29.79 μg/ml, respectively. The *C*_max_ was highest in the kidney and lowest in adipose and brain. The *T*_max_ of muscle and liver was 1.5 and 1 h, respectively; for other tissues, 1.25 h.

### Parameter Sensitivity Analysis

**FIGURE 9:**
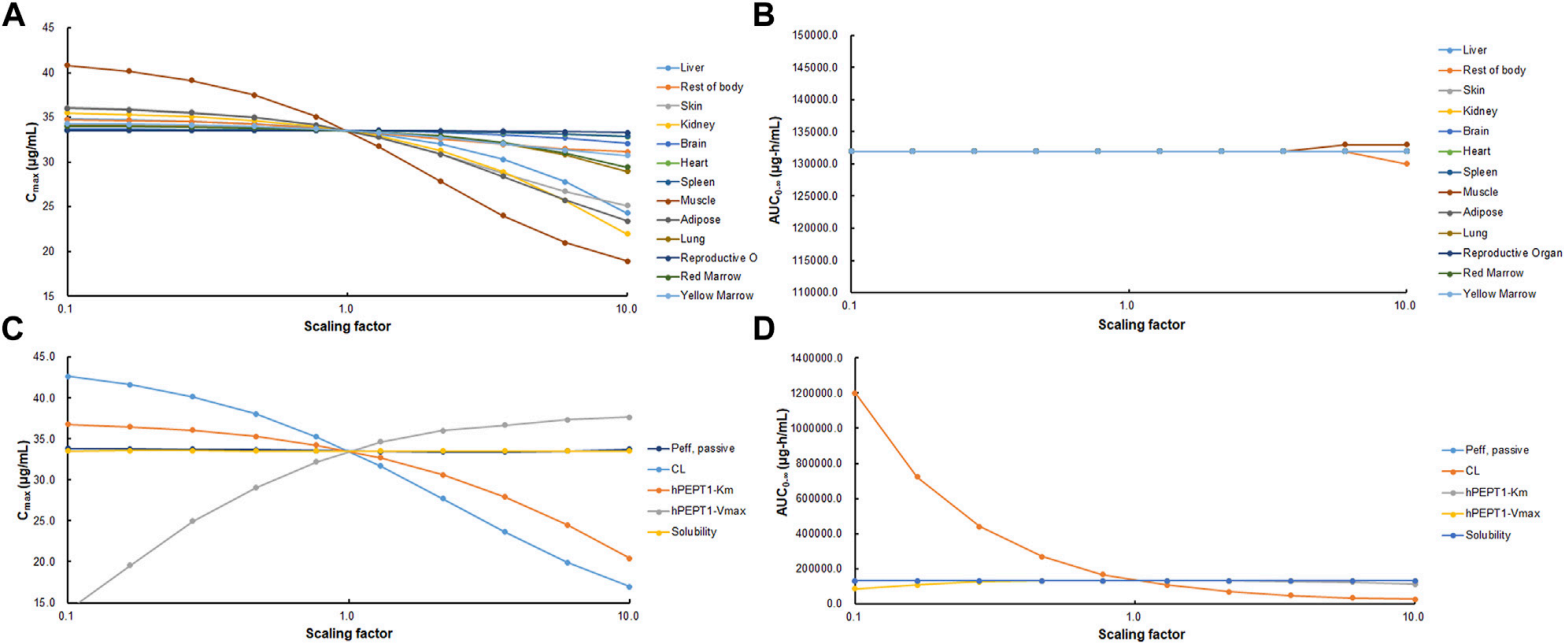
Sensitivity of predicted *C*_max_ **(A, C)** and *AUC*_0-∞_ **(B, D)** of cefadroxil using physiologically based pharmacokinetic (PBPK) model in human. Parameters were varied by multiplying the initial input values with scaling factors in the range of 0.1–10.

As shown in Figures 9A and B, PSA suggested that the predicted *C*_max_ of cefadroxil was most sensitive to changes in muscle *K*_p_. It was also sensitive to changes in *K*_p_ of kidney, adipose, and liver. The changes in *K*_p_ values almost do not cause the changes in *AUC*_0-∞_. As shown in Figures 9C and D, PSA indicated that the predicted *C*_max_ and *AUC*_0-∞_ of cefadroxil were sensitive to changes in clearance in kidney and *V*_max_ of hPEPT1. However, they were insensitive to passive permeability and solubility of cefadroxil.

### Effect of hPEPT1 on the Oral Absorption of Cefadroxil

**FIGURE 10:**
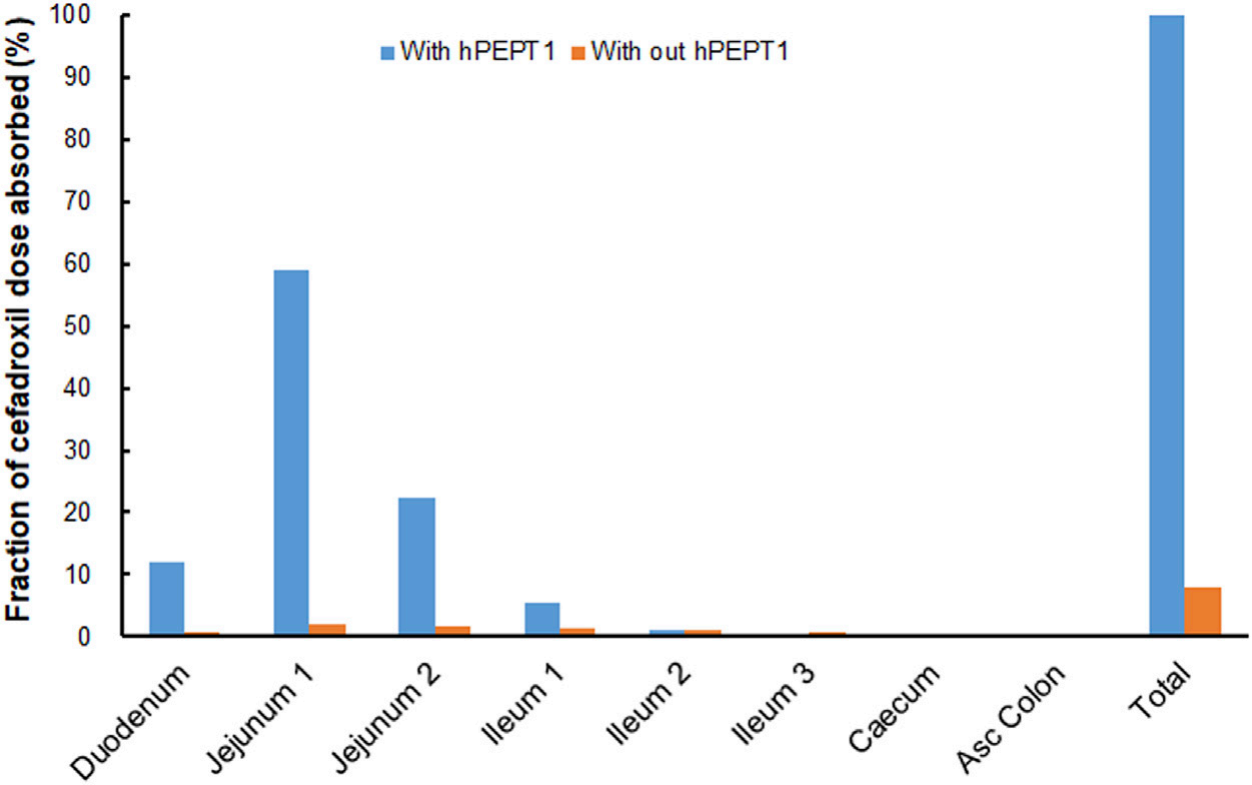
Contribution of specific human intestinal regions in the oral absorption of 15 mg/kg cefadroxil (with and without hPEPT1).

**FIGURE 11:**
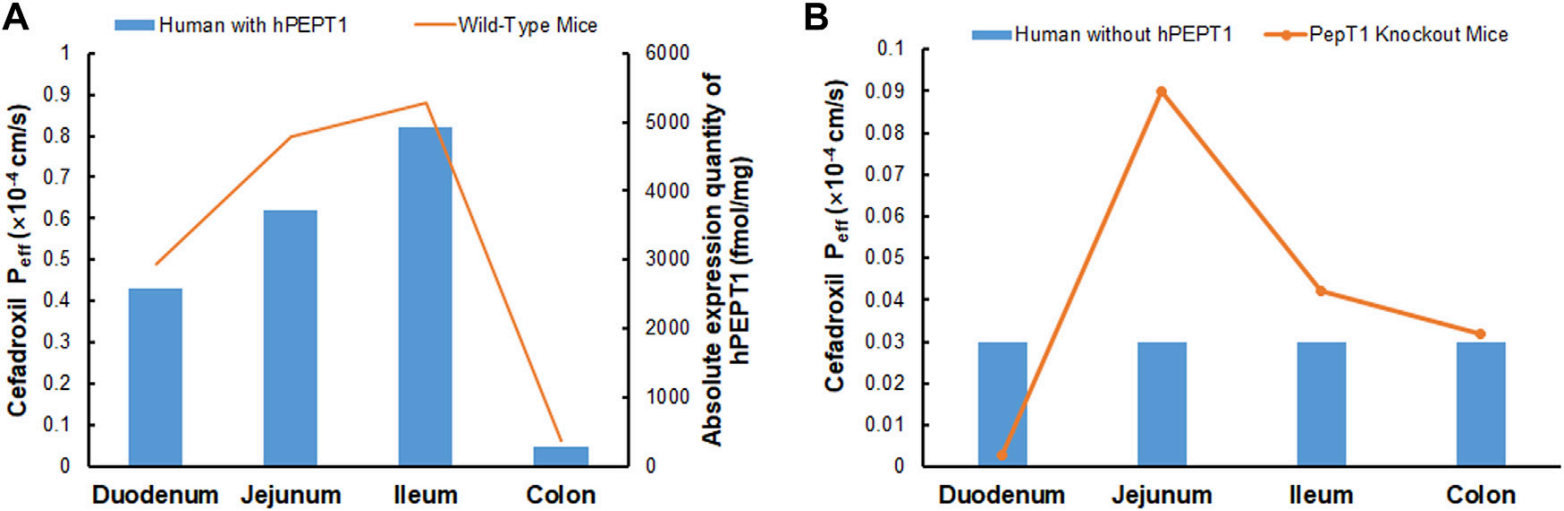
Absolute expression quantity of hPEPT1 in human and effective permeability of cefadroxil in wild-type mouse duodenum, jejunum, ileum, and colon (n = 4–8) **(A)**. The passive permeability of cefadroxil in human and effective permeability of cefadroxil in PepT1 knockout mouse duodenum, jejunum, ileum, and colon (n = 4–8) **(B)**.

The simulated fractions of cefadroxil dose absorbed in the absence and presence of hPEPT1 intestinal expression are shown in Figure 10. The fraction of cefadroxil dose absorbed was 7.8% in the absence of hPEPT1 and 99.9% in the presence of hPEPT1. As shown in Figure 11A, there is a good correlation between the absolute expression quantity of hPEPT1 in human and cefadroxil effective permeability in wild-type mouse duodenum, jejunum, ileum, and colon. The humans and mice have large absorption at duodenum, jejunum, and ileum, but their absorptions at colon were both small. The passive permeability used in the PBPK model in human and effective permeability of cefadroxil in PepT1 knockout mice were shown in Figure 11B. The values were both much less than the absorption through hPEPT1.

### Effect of Drug Release Rate on the Oral Absorption of Cefadroxil

The predicted plasma concentration–time profiles of cefadroxil when using different dissolution rates are shown in Supplementary Figure S2. The relative errors of *C*_max_ and *AUC*_0-∞_ were within 20% up to T_85%_ = 2 h. This indicated that the *C*_max_ and *AUC*_0-∞_ were not sensitive to dissolution rate when the dissolution rate was not slower than T_85%_ = 2 h.

## Discussion

### The *K*_p_ Values Used in the Physiologically Based Pharmacokinetic Models

As shown in Table 3, there are some discrepancies between predicted *K*_p_ values in human and observed values in mouse and rat, especially for kidney. Cefadroxil was excreted primarily by the kidney, with over 90% of the administered dose being recovered in the urine intact within 24 h. PEPT2 (SLC15A2) mediates the renal reabsorption of cefadroxil (Xie et al., 2016). The predicted method cannot consider this factor and led to the discrepancy. The observed *K*_p_ values in mouse and rat are closer to most tissues.

The incomplete observed tissue distribution data in human were collected as below. The mean cefadroxil concentration ratios of gingiva/serum and mandibular bone/serum at the peak time were 0.54 and 0.21 (Akimoto et al., 1994). The concentrations of cefadroxil in saliva 3–4 h after drug administration were 40%–112% of those found in serum. The peak concentrations of cefadroxil in pleural fluid after a single 1-g dose of cefadroxil in 4 patients were about 50% of those found in serum. The peak concentration of cefadroxil in lung tissue was 54%–69% of the serum value (Nightingale, 1986). The mean value of *C*_bone_/*C*_plasma_ at the same time from administration was 0.3 (Nungu et al., 1995). The peak concentration of cefadroxil in skin blister was 20 mg/L after 3 h. The peak concentration of cefadroxil in serum was 28.4 mg/L after 1.5 h (Simon et al., 1980). The cefadroxil tissue concentrations in hepatobiliary tissues were roughly parallel of those seen in plasma (Palmu et al., 1980). The *K*_p_ values of tissues used in PBPK model were almost consistent with these observed distribution data in human. This further validated the reliability of *K*_p_ values used in the PBPK models and the reasonability of the PBPK models in this study.

### Parameter Sensitivity Analysis

Muscle tissue accounted for 33.52% of the *BW* and had relatively high blood flow. This induced that *C*_max_ of cefadroxil was most sensitive to changes in muscle *K*_p_. Adipose accounted for 32.37% of *BW*, so it also affected the *C*_max_ of cefadroxil. Kidney and liver were quick blood perfusion organs; besides, the kidney was the elimination organ in this study. Therefore, the *C*_max_ of cefadroxil was sensitive to changes in their *K*_p_. For these four tissues and organs, the observed and reasonable *K*_p_ values were used in this study. Though the *C*_max_ and *AUC*_0-∞_ of cefadroxil were not sensitive to some tissue *K*_p_ values, the tissue *K*_p_ values were important to assess the distribution of the drug in the tissues.

The solubilities of cefadroxil in water from pH 3 to 6.88 were added in the PBPK models (Shoghi et al., 2013). Cefadroxil was quite soluble among these pH values, so its *C*_max_ and *AUC*_0-∞_ values were insensitive to solubility. Abundant hPEPT1 expressed along the intestine plays a major role in the absorption of cefadroxil. This makes the *C*_max_ and *AUC*_0-∞_ values insensitive to passive permeability but sensitive to *V*_max_ of hPEPT1.

### Model Calibration

The clearances in the PBPK models of mouse, rat, and human in this study were obtained by optimizing the value based on observed plasma concentration–time curves. For the PBPK model of mouse and human, the calibrations were both based on one dosage and validated by the other two dosages. For the PBPK model of rat, the calibration was based on one dosage. The *K*_p_ values of adipose, reproductive organ, and rest of body were calibrated based on the predicted *K*_p_ values using GastroPlus™, observed plasma concentration–time curves, and experience. The *V*_ss_ values calculated by PBPK models were almost identical with the values published in the observed PK articles. The *V*_ss_ and *CL* values among the three species were in the good allometric scaling relationship. These all indicated the reasonability of the calibration.

### Effect of hPEPT1 on the Oral Absorption of Cefadroxil

The fraction of cefadroxil dose absorbed decreased from 99.9% to 7.8% when the simulations were performed without hPEPT1. Cefadroxil was absorbed completely with hPEPT1 among these three dosages when simulating using PBPK model in human in this study. PepT1 ablation resulted in 23-fold reductions in peak plasma concentrations and 14-fold reductions in systemic exposure of cefadroxil after oral dosing in wild-type and PepT1 knockout mice (Posada and Smith, 2013b). These both demonstrated that PepT1 is the major transporter responsible for the oral absorption of cefadroxil.

### Effect of Drug Release Rate on the Oral Absorption of Cefadroxil

The *C*_max_ and *AUC*_0-∞_ of cefadroxil were insensitive to the dissolution rate up to T_85%_ = 2 h because of the high solubility of cefadroxil. When the dissolution rates were slower than T_85%_ = 2 h, more drug would be released at the lower segment of the intestine, where the expression of hPEPT1 is smaller. This induces to a decrease in oral absorption of cefadroxil. In order to reduce administration times per day and increase patient compliance, cefadroxil was often designed as a sustained-release dosage form. This study suggested that attention should be paid to the oral absorption of cefadroxil when the release rate of its formulation is too slow.

### The Limitations of This Study

There are some limitations of this study due to the assumptions and data sources. The plasma protein-binding data obtained from Drugbank database were used in PBPK models of all the species in this study. The data in each species were not found. There may exist in the blood–brain barrier for cefadroxil, but it was not considered due to lack of data.

Cefadroxil is a substrate of PEPT2 (SLC15A2), and PEPT2 mediates the renal reabsorption of cefadroxil (Xie et al., 2016). The renal clearances of mouse, rat, and human were 0.52, 3.00, and 141.67 ml/min in this study. They were slightly higher than the glomerular filtration rates (GFRs) of these three species (mouse: about 0.30 ml/min, rat: about 2.38 ml/min, human: about 125 ml/min), indicating that there may be reabsorption and active secretion. These were complicated and were not considered in the PBPK models in the study, instead just the *CL* was added in the kidney. For cefadroxil, there was no evidence of its nonlinear intestinal absorption in mice (Posada and Smith, 2013a). However, there was an increase in plasma clearance as the dose increases in rat. This phenomenon was attributed to a saturable renal tubular reabsorption (Garcia-Carbonell et al., 1993). The PK behavior of cefadroxil was dose-dependent in human, too. This phenomenon was the result of the combined action of saturable active gastrointestinal absorption (PEPT1) and saturable renal tubular reabsorption of cefadroxil (PEPT2) (Garrigues et al., 1991). The PBPK models in this study considered the PEPT1, but did not consider the PEPT2, thus this no-linear phenomenon was not found in rat and human in our PBPK models.

## Conclusion

In the present study, the PBPK models in mouse, rat, and human simulating the plasma and tissue concentration–time profiles of cefadroxil were established successfully and validated strictly by the observed PK data. The models’ rationality and accuracy were further demonstrated by the almost consistent *V*_ss_ calculated by different methods, good allometric scaling relationship of *V*_ss_ and *CL*, and model PSA. The PBPK model in human suggested that hPEPT1 was the major transporter responsible for the oral absorption of cefadroxil in human. It also suggested that the plasma concentration–time profile of cefadroxil was not sensitive to dissolution rate faster than T_85%_ = 2 h. All in all, the PBPK model in human may be useful for dose selection and informative decision-making during clinical trials and dosage form design of cefadroxil and provide a reference for the PBPK studies of hPEPT1 substrate.

## Data Availability

The original contributions presented in the study are included in the article/Supplementary Material, further inquiries can be directed to the corresponding author.

## Supplementary Material

Supplementary Figure S1 Predicted tissues and plasma concentration-time profiles of cefadroxil in human after oral administration 15 mg/kg. The dots were the observed data in plasma.

Supplementary Figure S2 The effect of release rate on the simulated plasma concentration-time profiles of cefadroxil after oral administration of 15 mg/kg cefadroxil in human.

Supplementary Table S1 The predicted tissues and plasma concentrations of cefadroxil after oral dosing 15 mg/kg obtained from the PBPK model in human in this study.
