# Supplementary material for: Physiologically Based Pharmacokinetic Modeling of Cefadroxil in Mouse, Rat, and Human to Predict Concentration–Time Profile at Infected Tissue
Source: Front Pharmacol. 2021 Dec 23;12:692741. doi: 10.3389/fphar.2021.692741 (PMC8733657; doi:10.3389/fphar.2021.692741)
Supplement: Supplementary file 1 [file Table1.docx]

**Supplementary Table S1 |** The predicted tissues and plasma concentrations of cefadroxil after oral dosing 15 mg/kg obtained from the PBPK model in human in this study

| Time(h) | Concentrations (μg/mL) | | | | | | | | | | | |
| --- | --- | --- | --- | --- | --- | --- | --- | --- | --- | --- | --- | --- |
|  | Lung | Plasma | Adipose | Muscle | | Liver | | Spleen | Heart | Brain | Kidney | Skin |
| 0.17 | 2.30 | 4.49 | 0.20 | | 0.36 | | 9.65 | 1.62 | 0.92 | 0.45 | 19.78 | 1.38 |
| 0.33 | 5.77 | 11.16 | 0.76 | | 1.53 | | 17.57 | 4.33 | 2.44 | 1.18 | 59.56 | 5.76 |
| 0.5 | 9.32 | 17.98 | 1.43 | | 3.07 | | 24.11 | 7.13 | 4.02 | 1.93 | 101.51 | 11.38 |
| 0.75 | 13.99 | 26.96 | 2.38 | | 5.43 | | 31.01 | 10.86 | 6.10 | 2.93 | 158.28 | 19.81 |
| 1 | 17.08 | 32.86 | 3.10 | | 7.36 | | 33.37 | 13.38 | 7.51 | 3.60 | 198.05 | 26.59 |
| 1.25 | 17.80 | 34.23 | 3.40 | | 8.32 | | 31.10 | 14.05 | 7.88 | 3.77 | 210.22 | 29.79 |
| 1.5 | 16.93 | 32.54 | 3.32 | | 8.33 | | 27.52 | 13.40 | 7.51 | 3.59 | 201.63 | 29.63 |
| 2 | 14.39 | 27.66 | 2.85 | | 7.26 | | 22.66 | 11.40 | 6.39 | 3.05 | 171.79 | 25.70 |
| 3 | 10.31 | 19.82 | 2.04 | | 5.21 | | 16.20 | 8.17 | 4.58 | 2.19 | 123.09 | 18.42 |
| 4 | 7.40 | 14.21 | 1.47 | | 3.74 | | 11.62 | 5.86 | 3.28 | 1.57 | 88.26 | 13.21 |
| 6 | 3.80 | 7.31 | 0.75 | | 1.92 | | 5.98 | 3.01 | 1.69 | 0.81 | 45.40 | 6.79 |
| 8 | 1.96 | 3.76 | 0.39 | | 0.99 | | 3.08 | 1.55 | 0.87 | 0.42 | 23.38 | 3.50 |
