# Supplementary figures and images for: Physiologically Based Pharmacokinetic Modeling of Cefadroxil in Mouse, Rat, and Human to Predict Concentration–Time Profile at Infected Tissue
Source: Front Pharmacol. 2021 Dec 23;12:692741. doi: 10.3389/fphar.2021.692741 (PMC8733657; doi:10.3389/fphar.2021.692741)

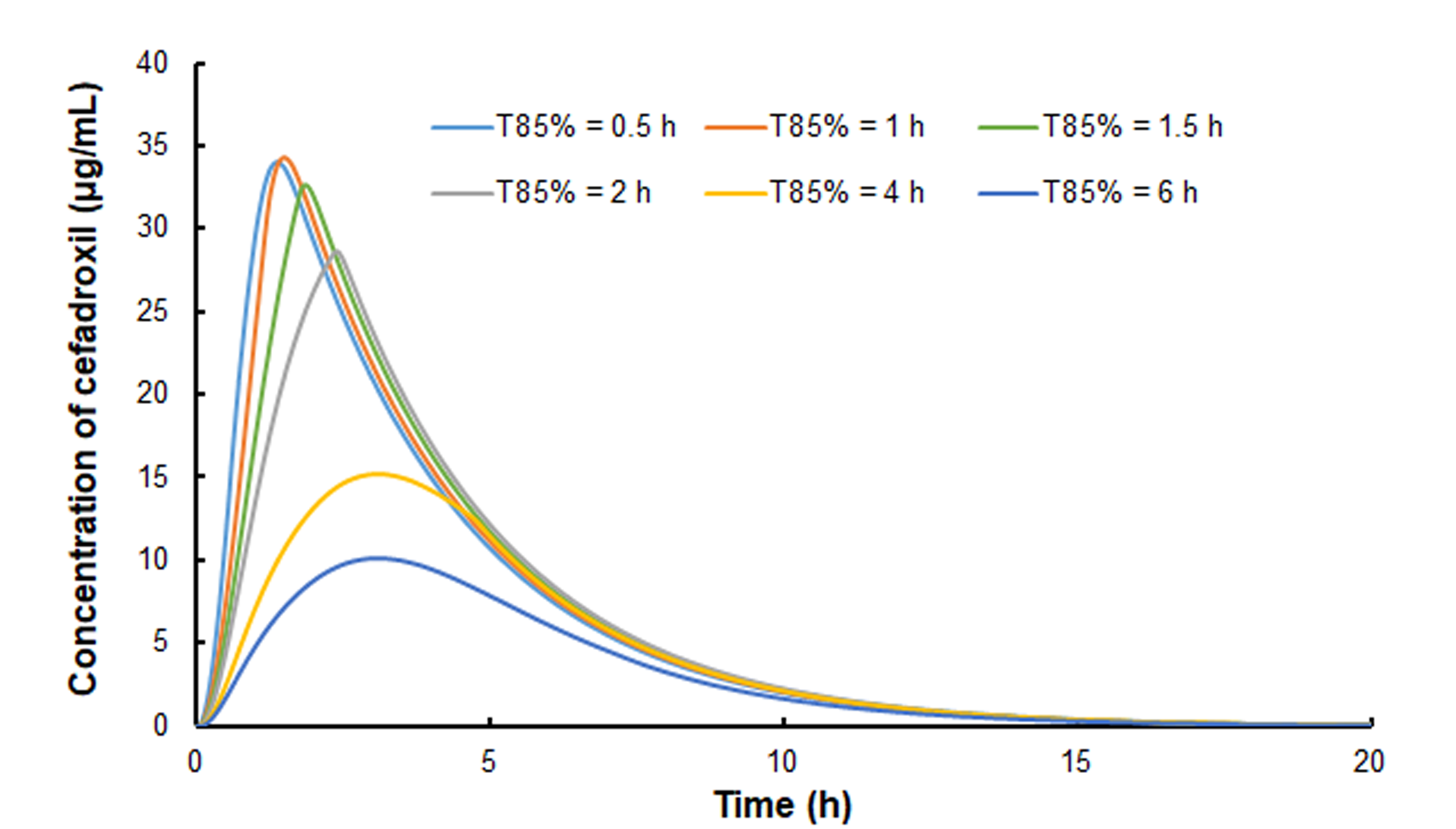

Supplement: Supplementary file 2 [file Image2.TIF]

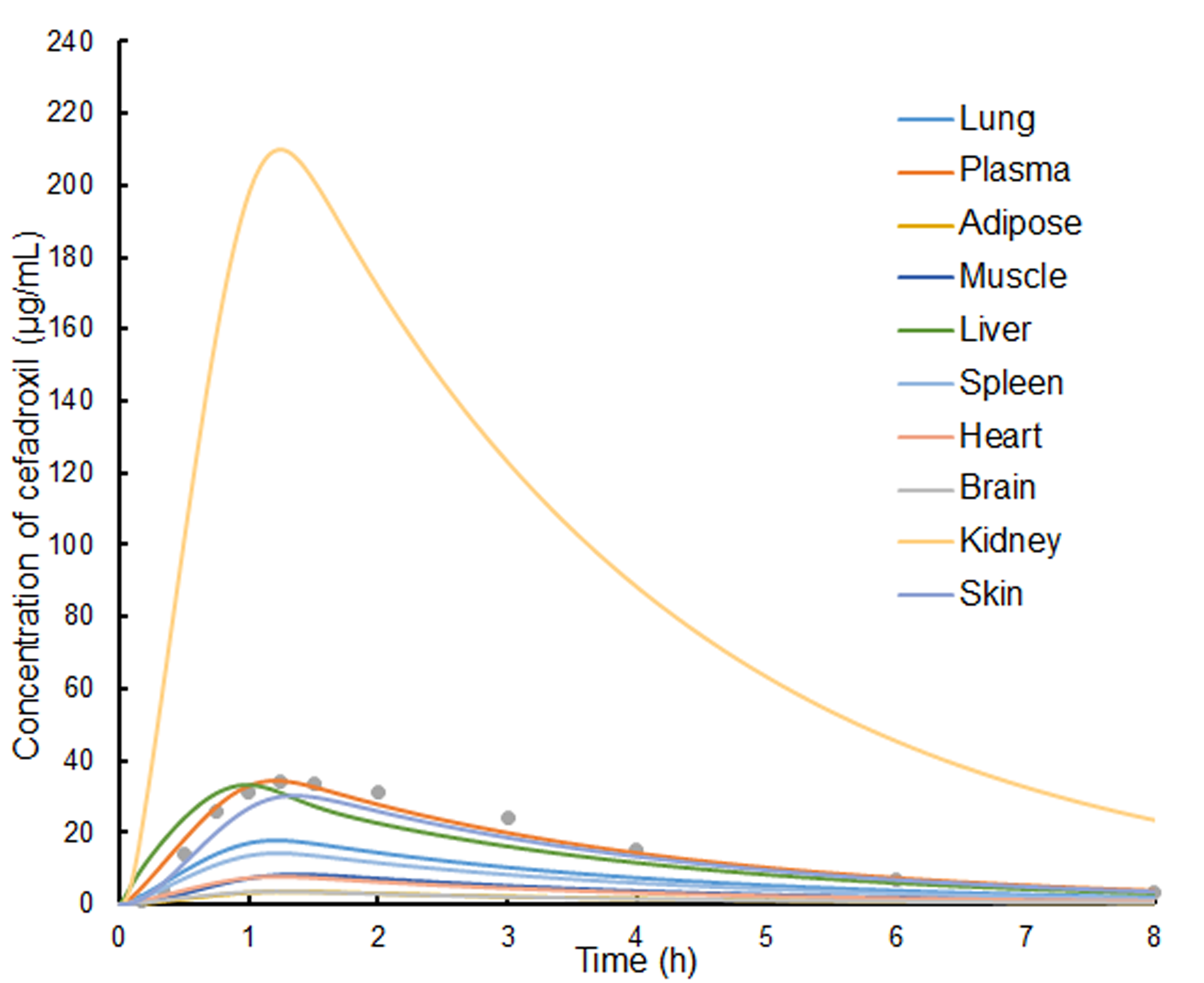

Supplement: Supplementary file 3 [file Image1.TIF]
